# Infection by Intestinal Parasites, Stunting and Anemia in School-Aged Children from Southern Angola

**DOI:** 10.1371/journal.pone.0137327

**Published:** 2015-09-15

**Authors:** Dinamene Oliveira, Filipa Santana Ferreira, Jorge Atouguia, Filomeno Fortes, António Guerra, Sónia Centeno-Lima

**Affiliations:** 1 Clínica Girassol, Lubango, Angola; 2 Global Health and Tropical Medicine, Unidade de Clínica Tropical, Instituto de Higiene e Medicina Tropical, Universidade Nova de Lisboa, Lisboa, Portugal; 3 Ministério da Saúde, Luanda, Angola; 4 Departamento de Pediatria, Faculdade de Medicina, Universidade do Porto, Porto, Portugal; University of Chicago, UNITED STATES

## Abstract

**Introduction:**

Intestinal parasites are responsible for morbidity in children worldwide, especially in low income countries. In the present study we determine the prevalence of intestinal parasites and explore its association with anemia and stunting in school-aged children.

**Methods:**

A cross-sectional study was conducted from September to October 2010 enrolling 328 children attending the primary school in Lubango, the second largest city after the capital Luanda. Stool samples were collected for parasite detection through microscopy and molecular identification of *Entamoeba histolytica* and *Entamoeba dispar*. Stunting was assessed using the z-scores of height for age and hemoglobin concentration was determined using a portable hemoglobin analyzing system.

**Results:**

The global prevalence of pathogenic intestinal parasites was 44.2%, the most common being *Ascaris lumbricoides* (22.0%), *Giardia lamblia* (20.1%) and *Hymenolepis nana* (8.8%). Molecular detection revealed that 13.1% of the children carried *E*. *dispar* and 0.3% were infected with *E*. *histolytica*. The prevalence of stunting (mild to severe) was 41.5%. Stunting was more frequent in older children (p = 0.006, OR = 1.886), while anemia was more frequent in younger children (p = 0.005, OR = 2.210). The prevalence of anemia was 21.6%, and we found a significant association with infection by *H*. *nana* (p = 0.031, OR = 2.449).

**Conclusions:**

This is one of the few published studies reporting intestinal parasites infection, nutritional status and anemia in children from Angola. Furthermore, the present work highlights the importance of regular intestinal parasites screening in children.

## Introduction

Intestinal parasites are responsible for morbidity and mortality worldwide, especially in low-income countries and in people with other diseases [1], and are more prevalent in hot and humid environments, with poor sanitation, contaminated water, poor housing and overcrowded [2]. Such environments are common in the suburbs of many African cities.

In sub-Saharan Africa, children, especially school-aged children, are disproportionately affected by soil-transmitted helminth infections [3]. In the study carried out in Mozambique, which was attended by 83331 children and youth from 7 to 22 years old from 1275 primary schools, were detected *Ascaris lumbricoides* (65.8%), *Trichuris trichiura* (54.0%), hookworms (38.7%), *Entamoeba* spp. (31.2%), *Giardia lamblia* (19.0%), *Taenia* spp. (5.8%) and *Hymenolepis nana* (5.2%) [4].

It is known that undernutrition may increase susceptibility to infection [5] while several studies have demonstrated an association between infection with intestinal parasites and undernutrition [6–8]. Therefore, infection with intestinal parasites and nutritional status influence each other in a vicious cycle, and it is difficult to establish the effect of each.

In addition to the nutritional status, iron deficiency anemia has also been associated with infection by intestinal parasites, namely *Schistosoma mansoni*, hookworms, *T*. *trichiura* and *A*. *lumbricoides* [9]. Anemia has complex etiological factors, including micronutrient deficiencies (iron, folate, riboflavin, vitamin A and B12), haemoglobinopathies and parasitic infections [10].

The present study aimed at determining the prevalence of intestinal parasites in children from 5 to 12 years old attending primary school, in the community of Lucrécia, Lubango, Angola, in September and October of 2010 and explored the possible relationships between intestinal parasite infection, stunting, and anemia.

## Methods

### Study Design

A cross-sectional study was conducted between September and October 2010 in the 3 public primary schools in the community of Lucrécia, Lubango city, Huíla Province, Angola. The study was carried out in collaboration with local health and education authorities, including the Provincial Direction of Education and the Provincial Direction of Health.

### Study Population and Sampling

The study population were children aged 5 to 12 years, attending the 3 public primary schools in the community of Lucrécia, Lubango, Angola: Escola Primária N°194 with 1590 children, Escola Primária Abrigo Anjo da Guarda with 382 children and Escola 1° de Dezembro with 651 children, with a total population of 2623 children (N). Sample size was calculated using the following formula: n ≥ ⌈[*Z* ^2^p(1 − p)]/[Δ^2^]⌉, where n is the required sample size, Z (0.975 quantile of a normal distribution for a confidence interval of 95%) = 1.96, p is the prevalence of intestinal parasites in the population which, being unknown, was considered 0.5 and Δ (error) = 0.05. The value was corrected, as follows: n′ = n × [(N − n)/(N − 1)]. The estimated minimum size of the sample was 329 children. Data from 329 children were collected. However, due to the fact that one of them has less than 5 years, 328 children were enrolled.

### Parasitological Diagnosis

The teacher gave to each child parents/guardians a sterile container and explained the procedure for the collection of a single stool sample. A portion of each fresh stool sample was stored in a Eppendorf tube with a storage and transport liquid, Protofix (*Alexon-Trend*, *Inc*.), and another portion was conserved in filter paper (*Generation® Blood Collection Card*, *Qiagen*) for subsequent molecular analysis.

Parasite detection and identification in the stools was conducted through microscopic examination of the samples in iodine [11] at the Institute of Hygiene and Tropical Medicine in Lisbon, Portugal. Each sample was observed in triplicate by two trained microscopists. No concentration technique was applied.

### Molecular Analysis

DNA was extracted from all samples in which *Entamoeba histolytica/dispar* cysts were detected by microscopy [12–13]. Molecular identification of *E*. *histolytica* and *E*. *dispar* DNA was conducted as described elsewhere [14] with 40 amplification cycles. Additionally an internal amplification control was also performed in all samples as described previously [15], with 40 amplification cycles in the second amplification reaction.

### Anthropometry

Height was measured with a wall stadiometer Seca 208 (precision of 0.1 cm) with child head positioned according to the Frankfurt plane. Z-scores of height for age were calculated using the WHO AnthroPlus software. The height for age is an indicator of stunting, classified as mild (z-scores<-1 and ≥-2), moderate (z-scores<-2 and ≥-3) and severe (z-scores<-3) [16].

### Anemia Diagnosis

A single measurement of hemoglobin concentration in each child was conducted using a portable hemoglobin analyzing system HB 301+ (*HemoCue® AB*, *Angelhome*, *Sweden*). Classification of anemic or non-anemic children, and subsequently in mild, moderate and severe anemia, was performed according to the reference values adjusted for the altitude [17] (Table 1) as Lubango is 1786 meters above sea level [18].

**Table 1:** Reference values for anemia diagnose (g/dl).

Age (years)	Not Anemic	Mild Anemia	Moderate Anemia	Severe Anemia
**5–11**	≥11.5	11.0–11.4		
			8.0–10.9	<8.0
**12**	≥12.0	11.0–11.9		

### Children Treatment

The treatment of infected children was prescribed by a specialist physician in infectious diseases and tropical medicine. The National Program for Control of Neglected Diseases provided the drugs that were given to parents/guardians after a short explanation by local doctors of the Pediatric Hospital of Lubango.

### Statistical Methodology

A database was constructed using SPSS (version 19). Association between two qualitative variables was explored using Chi-square test or the Fisher's Exact test whenever required.

The multivariate regression models were applied for the dependent variables, stunting and anemia, included the independent variables for which a statistically significant association was found by binary regression (*p*<0.05).

### Ethical Considerations

This study was approved by the Ministry of Health in Angola and by the Ethic Committee of IHMT in Portugal, as well as by the Province Health and Education Authorities. Only children whose parents/guardians signed the informed consent were included. Whenever the parents/guardians could not read or write, one of the team members read aloud and then required the fingerprint and a signature by a witness.

## Results

The 328 children were aged between 5.43 and 12.98 years (mean age of 9.61 and SD of 2.02). 52.8% (173/328) were less than 10 years and females represented 56.4% (185/328) of the enrolled participants.

Stool sample analysis revealed that 44.2% (145/328) of the children were infected with at least one species of pathogenic intestinal parasite. Helminths were more frequent than protozoa infecting 24.1% (79/328) vs 13.4% (44/328) of the studied children. Coinfection by protozoa and helminths occurred in 6.7% (22/328) of the enrolled participants. In parallel single infections were more frequent (35.7%, 117/328) than coinfections (8.5%, 28/328). Globally, the most prevalent parasite was *A*. *lumbricoides* either in single or mixed infections, detected in 22.0% (72/328) of the children, followed by *G*. *lamblia* with 20.1% (66/328) and *H*. *nana* with 8.8% (29/328) (Table 2). DNA of *E*. *histolytica* was detected in one sample (0.3%) while 43 of the children were carrying *E*. *dispar* DNA (13.1%). No statistically significant difference in intestinal parasite prevalence was observed either for sex or age (Table 3).

**Table 2:** Type of infection by pathogenic intestinal parasite: relative frequencies (%), absolute frequencies (n) and confidence intervals of 95% (IC95%).

Parasite		% (n)	IC95%
	***A*. *lumbricoides***	16.2 (53)	(12.2;20.2)
	***G*. *lamblia***	13.4 (44)	(9.7;17.1)
	***H*. *nana***	4.6 (15)	(2.3;6.9)
**Monoinfection**	***Taenia* spp.**	0.6 (2)	(0.0;1.4)
	**Hookworms**	0.6 (2)	(0.0;1.4)
	***S*. *stercoralis***	0.3 (1)	(0.0;0.9)
	**Total**	35.7 (117)	(30.5;40.9)
**Coinfection**	***A*. *lumbricoides* and *G*. *lamblia***	3.4 (11)	(1.4;5.4)
	***H*. *nana* and *G*. *lamblia***	1.8 (6)	(0.4;3.2)
	***A*. *lumbricoides* and *H*. *nana***	0.9 (3)	(0.0;1.9)
	***A*. *lumbricoides*, *H*. *nana* and *G*. *lamblia***	0.6 (2)	(0.0;1.4)
	***H*. *nana* and Hookworms**	0.6 (2)	(0.0;1.4)
	***A*. *lumbricoides*, *T*. *trichiura* and *G*. *lamblia***	0.3 (1)	(0.0;0.9)
	***H*. *nana*, Hookworms and *G*. *lamblia***	0.3 (1)	(0.0;0.9)
	***A*. *lumbricoides* and *Taenia* spp.**	0.3 (1)	(0.0;0.9)
	***A*. *lumbricoides*, *G*. *lamblia* and *E*. *histolytica***	0.3 (1)	(0.0;0.9)
	**Total**	8.5 (28)	(5.5;11.5)
**No Infection**		55.8 (183)	(50.4;61.2)
**Total**		100.0 (328)	

**Table 3:** Binary regression for infection by pathogenic intestinal parasite.

	Infection by Pathogenic Intestinal Parasite			
	% (n)	*p*	OR	IC95% (OR)
**Age Group (years)**		0.581^a^		
5–10 (n = 173)	42.8 (74)		(ref.)	—-
10–12 (n = 155)	45.8 (71)		1.131	(0.731;1.750)
**Gender**		0.961^a^		
Female (n = 185)	44.3 (82)		1.011	(0.651;1.569)
Male (n = 143)	44.1 (63)		(ref.)	—-

% (n)–relative and absolute frequencies of infected children; *p*—refers to the comparison of the ratio of the variable classes; OR—Odds Ratio; IC95% (OR)—confidence interval at 95% of the estimated odds ratio; (ref.)—reference category for OR

a–Pearson Chi-square test

The prevalence of stunting was 41.5% (136/328) and was mostly mild. Anemia was detected in 21.6% (71/328) of children and was most frequently moderate. No cases of severe anemia were identified (Table 4).

**Table 4:** Level of stunting and anemia (n = 328): relative frequencies (%), absolute frequencies (n) and confidence intervals of 95% (IC95%).

	Stunting		Anemia	
Level	% (n)	IC95%	% (n)	IC95%
**Mild**	32.0 (105)	(27.0; 37.0)	9.7 (32)	(6.5;12.9)
**Moderate**	8.3 (27)	(5.3;11.3)	11.9 (39)	(8.4;15.4)
**Severe**	1.2 (4)	(0.0; 2.4)	0.0 (0)	—-

Demographic characteristics and infections by pathogenic intestinal parasites were used as independent variables in binary regression for stunting and anemia. Stunting was associated to age, type of infection, infection by *H*. *nana*, *A*. *lumbricoides* (in coinfection) and *G*. *lamblia* (in coinfection). Age and infection by *H*. *nana* (total and in coinfection) were significantly associated with anemia (Table 5).

**Table 5:** Binary regression for stunting and anemia.

	Stunting				Anemia			
	% (n)	*p*	OR	IC95% (OR)	% (n)	*p*	OR	IC95% (OR)
**Age Group (years)**		**0.008** ^a^				**0.005** ^a^		
5–10 (n = 173)	34.7 (60)		(ref.)	**—-**	27.7 (48)		**2.204**	**(1.266;3.835)**
10–12 (n = 155)	49.0 (76)		**1.812**	**(1.162;2.825)**	14.8 (23)		(ref.)	**—-**
**Gender**		0.197^a^				0.580^a^		
Female (n = 185)	38.4 (71)		(ref.)	**—-**	20.5 (38)		(ref.)	**—-**
Male (n = 143)	45.5 (65)		1.338	(0.859; 2.084)	23.1 (33)		1.161	(0.685;1.967)
**Infection by Pathogenic Intestinal Parasite**		0.271^a^				0.708^a^		
No (n = 183)	38.8 (71)		(ref.)	**—-**	22.4 (41)		(ref.)	**—-**
Yes (n = 145)	44.8 (65)		1.282	(0.824;1.994)	20.7 (30)		0.903	(0.531;1.537)
**Type of Parasite**		0.166^a^				0.442^a^ ^1^		
No Parasite (n = 183)	38.8 (71)		(ref.)	**—-**	22.4 (41)		(ref.)	**—-**
Protozoa (n = 44)	43.2 (19)		1.199	(0.616 ;2.335)	22.7 (10)		1.019	(0.464;2.236)
Helminths (n = 79)	40.5 (32)		1.074	(0.627;1.841)	16.5 (13)		0.682	(0.343;1.358)
Protozoa and Helminths (n = 22)	63.6 (14)		**2.761**	**(1.102;6.914)**	31.8 (7)		1.616	(0.618;4.230)
**Type of Infection**		**0.036** ^a^				0.093^a^		
No infection (n = 183)	38.8 (71)		(ref.)	**—-**	22.4 (41)		(ref.)	**—-**
Monoinfections (n = 117)	40.2 (47)		1.059	(0.659;1.702)	17.1 (20)		0.714	(0.394;1.293)
Coinfections (n = 28)	64.3 (18)		**2.839**	**(1.240; 6.500)**	35.7 (10)		1.924	(0.824;4.491)
***A*. *lumbricoides* (total)**		0.756^a^				0.246^a^		
No (n = 256)	41.0 (105)		(ref.)	**—-**	23.0 (59)		(ref.)	**—-**
Yes (n = 72)	43.1 (31)		1.087	(0.641;1.845)	16.7 (12)		0.668	(0.337;1.324)
***A*. *lumbricoides* (monoinfection)**		0.226^a^				0.103^a^		
No (n = 275)	42.9 (118)		(ref.)	**—-**	23.3 (64)		(ref.)	**—-**
Yes (n = 53)	34.0 (18)		0.684	(0.369;1.268)	13.2 (7)		0.502	(0.216;1.166)
***A*. *lumbricoides* (coinfection with other parasites)**		**0.014** ^a^				0.574^b^		
No (n = 309)	39.8 (123)		(ref.)	**—-**	21.4 (66)		(ref.)	**—-**
Yes (n = 19)	68.4 (13)		**3.276**	**(1.213;8.851)**	26.3 (5)		1.315	(0.457;3.783)
***G*. *lamblia* (total)**		0.115^a^				0.364^a^		
No (n = 262)	39.3 (103)		(ref.)	**—-**	20.6 (54)		(ref.)	**—-**
Yes (n = 66)	50.0 (33)		1.544	(0.897;2.656)	25.8 (17)		1.336	(0.713;2.503)
***G*. *lamblia* (monoinfection)**		0.804^a^				0.852^a^		
No (n = 284)	41.2 (117)		(ref.)	**—-**	21.5 (61)		(ref.)	**—-**
Yes (n = 44)	43.2 (19)		1.085	(0.571;2.061)	22.7 (10)		1.075	(0.503;2.299)
***G*. *lamblia* (coinfection with other parasites)**		**0.029** ^a^				0.281^b^		
No (n = 306)	39.9 (122)		(ref.)	**—-**	20.9 (64)		(ref.)	**—-**
Yes (n = 22)	63.6 (14)		**2.639**	**(1.075;6.481)**	31.8 (7)		1.765	(0.690;4.511)
***H*. *nana* (total)**		**0.049** ^a^				**0.026** ^a^		
No (n = 299)	39.8 (119)		(ref.)	**—-**	20.1 (60)		(ref.)	**—-**
Yes (n = 29)	58.6 (17)		2.143	(0.988;4.649)	37.9 (11)		**2.434**	**(1.092;5.427)**
***H*. *nana* (monoinfection)**		0.339^a^				1.000^b^		
No (n = 313)	40.9 (128)		(ref.)	**—-**	21.7 (68)		(ref.)	**—-**
Yes (n = 15)	53.3 (8)		1.652	(0.584;4.669)	20.0 (3)		0.901	(0.247;3.283)
***H*. *nana* (coinfection with other parasites)**		0.076^a^				**0.003** ^b^		
No (n = 314)	40.4 (127)		(ref.)	**—-**	20.1 (63)		(ref.)	**—-**
Yes (n = 14)	64.3 (9)		2.650	(0.868;8.092)	57.1 (8)		**5.312**	**(1.779;15.862)**

% (n)–relative and absolute frequencies of stunted or anemic children; *p*—refers to the comparison of the ratio of the variable classes; OR—Odds Ratio; IC95% (OR)—confidence interval at 95% of the estimated odds ratio

^a^–Pearson Chi-square test

^b^—Fisher's Exact test; (ref.)—reference category for OR

1—test with robustness problems, 12.5% of the expected frequencies below 5

Multivariate regression models to explain stunting and anemia were defined using the variables that shown to be significantly associated (*p*<0.05) in the binary analysis.

According to the multivariate regression model for stunting, only age was statistically significant (*p* = 0.006). Stunting was more frequent in children aged 10 to 12 years when compared with younger children (OR:1.886; IC95%(OR):1.199–2.967), adjusting for infections (Table 6).

**Table 6:** Multivariate regression model for stunting.

					Model Evaluation		
	% (n)	OR Adjusted	IC95% (OR Adjusted)	*p* ^a^	R^2^ (%)	*p* ^b^	*p* ^c^
					6.5	0.012	0.889
**Age Group (years)**				**0.006**			
5–10	34.7 (60)	(ref.)	—-	—-			
10–12	49.0 (76)	**1.886**	**(1.199;2.967)**	**0.006**			
**Type of Infection**				0.962			
No Infection	38.8 (71)	(ref.)	—-	—-			
Monoinfections	40.2 (47)	0.940	(0.570;1.553)	0.810			
Coinfections	64.3 (18)	1.107	(0.098;12.528)	0.935			
***A*. *lumbricoides* (coinfection with other parasites)**				0.290			
No	39.8 (123)	(ref.)	—-	—-			
Yes	68.4 (13)	2.675	(0.433;16.542)	0.290			
***G*. *lamblia* (coinfection with other parasites)**				0.968			
No	39.9 (122)	(ref.)	—-	—-			
Yes	63.6 (14)	1.042	(0.140;7.770)	0.968			
***H*. *nana* (total)**				0.215			
No	39.8 (119)	(ref.)	—-	—-			
Yes	58.6 (17)	1.903	(0.688;5.259)	0.215			

% (n)–relative and absolute frequencies of stunted children; OR Adjusted—Odds Ratio Adjusted for confounders; IC95% (OR Adjusted)—confidence interval at 95% of the estimated adjusted odds ratio

*p* ^a^—*p* refers to test the significance of each parameter levels in each factor (H_0_:OR = 1, hypothesis of no association); R^2^ (%)—percentage coefficient of determination of the model according to Nagelkerke

*p* ^b^—*p* refers to the significance level of the Omnibus test of model coefficients

*p* ^c^—*p* refers to the significance level of the Hosmer and Lemeshow test on the good fit of the model; (ref.)—reference category for OR

In the multivariate regression model for anemia, adjusted for the age group and infection by *H*. *nana*, both variables were statistically significant (*p* = 0.005 e *p* = 0.031, respectively) (Table 7).

**Table 7:** Multivariate regression model for anemia

					Model Evaluation		
	% (n)	OR Adjusted	IC95% (OR Adjusted)	*p* ^a^	R^2^ (%)	*p* ^b^	*p* ^c^
					5.8	0.002	0.423
**Age Group (years)**				**0.005**			
5–10	27.7 (48)	**2.210**	**(1.265; 3.861)**	**0.005**			
10–12	14.8 (23)	(ref.)	—-	—-			
***H*. *nana* (total)**				**0.031**			
No	20.1 (60)	(ref.)	—-	—-			
Yes	37.9 (11)	**2.449**	**(1.083; 5.536)**	**0.031**			

% (n)–relative and absolute frequencies of anemic children; OR Adjusted—Odds Ratio Adjusted for confounders; IC95% (OR Adjusted)—confidence interval at 95% of the estimated adjusted odds ratio

*p* ^a^—*p* refers to test the significance of each parameter levels in each factor (H_0_:OR = 1, hypothesis of no association); R^2^ (%)—percentage coefficient of determination of the model according to Nagelkerke

*p* ^b^—*p* refers to the significance level of the Omnibus test of model coefficients

*p* ^c^—*p* refers to the significance level of the Hosmer and Lemeshow test on the good fit of the model; (ref.)—reference category for OR

## Discussion

The analysis of stool samples revealed that 44.2% (145/328) of children in the study were infected with at least one type of pathogenic intestinal parasite. This prevalence is lower than the 80.0% found in the province of Bié, Angola, in a study where 791 children aged 6 to 10 years were enrolled [19]. The difference between the two prevalences can be related to the fact that non-pathogenic protozoa were included in the study from Bié [19].

In our study *A*. *lumbricoides* was the most prevalent soil-transmitted helminth (22.0%) while the prevalence of other helminths was very low, less than 2% (1.5% for Hookworms and 0.3% for *T*. *trichiura*). This could be related to the resistance of the eggs of *A*. *lumbricoides* to the type of soil in Lubango, a city located at 1786 meters altitude [18], as the eggs of *A*. *lumbricoides* can withstand over 2 years at temperature of 5–10°C [20]. For instance, in the Bengo Province, northwestern Angola, a study conducted only for soil-transmitted helminths in 1142 school-aged children (6–15 years old), from three communes (Caxito, Mabubas and Úcua) within the Dande municipality, found a prevalence of 31.6% [21].

The prevalence of *E*. *histolytica* worldwide remains unknown because very few studies use molecular methods, especially in Africa. The proportion of *E*. *histolytica*/*E*. *dispar* detected in the present study (1/43) highlights the importance of going beyond microscopy to identify the species of *Entamoeba*. In a study conducted in Gorgan city, located in northern Iran, 105 dysentery samples from children hospitalized in Taleghani hospital were collected and 25 were positive for *Entamoeba* complex in direct microscopic examination but PCR using positive controls indicated *E*. *histolytica and E*. *dispar* only in 2 and 3 samples, respectively [22]. As only *E*. *histolytica* is considered pathogenic [23], these findings reinforce the need to confirm the result of microscopy in order to avoid unnecessary treatment. This could be performed with molecular diagnosis in regions with adequate laboratory resources or through the detection of specific antigen (ELISA/RDT) in areas with poor laboratory resources.

Stunting represents the chronic state of undernutrition [24] that often begins in the uterus due to maternal undernutrition [25]. The majority of the studies related to undernutrition report data focused in children under 5 years of age. However, nutritional deficiencies in schoolchildren are also important as they compromise physical and cognitive development, and impact negatively on their learning ability [26]. Here we found that 41.5% (136/328) of the studied schoolchildren were stunted. Another research conducted in northern Angola revealed that 32.2% of the population aged between 6 months and 20 years were moderate or severely stunted [21].

In the present study stunting was associated to age group, being more frequent in children aged between 10 to 12 years compared to children less than 10 years, which may be related to the fact that older children have lived most troublesome times of food shortages in Angola associated with the period of war that ended in 2002.

The measurement of hemoglobin revealed that 21.6% (71/328) of children in the study had anemia. This value is slightly below the 29.7% from a screening carried out in Angola between 1998 and 1999 in 825 children less than 5 years of age [27]. This dissimilarity may be related to the difference in age groups between screenings, as is known that anemia is more prevalent in preschool-aged children [28].

The results from a survey of 2168 children aged ≤ 15 years in the Dande municipality in northern Angola suggest an association between *H*. *nana* infection and previous history of abdominal pain, and *H*. *nana* and *T*. *trichiura* coinfections to acute malnutrition in children aged ˂ 5 years [29]. Our results showed that *H*. *nana* was associated to anemia in the studied children, while another study found that mean value of hemoglobin (%), red blood cells and white blood cells counts and hematocrit (%) showed generalized decrease but without significant difference in *H*. *nana* patients and control denoting anemia liability [30]. In a survey that included preschool children from displacement camps, Khartoum state, Sudan, diarrhea was significantly associated with *H*. *nana* infection [31]. However, hymenolepiasis is absent from WHO guidelines for helminth control programs [29].

A limitation for this study may be related to the fact that only a single stool sample per child was analyzed and no concentration technique or Kato-Katz was applied, which may underestimate the prevalence of intestinal parasites. However, to minimize this situation each sample was observed in triplicate by two trained microscopists.

Almost half (44.2%) of studied children were infected with at least one type of pathogenic intestinal parasite. No statistically significant association between stunting and intestinal parasite infection was observed. A significant association between infection by *H*. *nana* and anemia was found. This results are particularly important in the context of health in Angola due to the scarcity of studies on this topic, providing information for the planning and implementation of a control program that links the fight against intestinal parasites, undernutrition and anemia.
